# Esophageal intramural squamous cell carcinomas presenting as subepithelial lesions removed by endoscopic submucosal dissection: Two case reports

**DOI:** 10.1097/MD.0000000000021850

**Published:** 2020-11-06

**Authors:** He Zhu, Bing Shi, Fudong Li, Hong Xu

**Affiliations:** Department of Gastroenterology, The First Hospital of Jilin University, ChangChun, Jilin, China.

**Keywords:** endoscopic submucosal dissection, intramural esophageal squamous cell carcinoma, subepithelial tumor

## Abstract

**Introduction:**

Esophageal subepithelial lesions (SELs) are rare, and the majority of them are benign. SELs are often covered with normal mucosa, thereby resulting in some malignant SELs to be easily missed or misdiagnosed. We report 2 cases of esophageal intramural squamous cell carcinomas (SCCs) that presented as SELs and were endoscopically removed.

**Patient concerns:**

Case 1 is a 63-year-old man with abdominal distension; case 2 is a 65-year-old man with increasing dysphagia for 2 months.

**Diagnosis:**

In case 1, endoscopy showed a 1.5-cm mucosal eminence with normal overlying mucosa. Endoscopic ultrasound (EUS) revealed that it might be derived from the muscularis mucosa or submucosa. In case 2, endoscopy revealed a 1.2-cm hemispherical lesion covered with smooth mucosa. Furthermore, EUS revealed that this lesion might be derived from the submucosa.

**Interventions:**

In both cases, the lesions were removed by endoscopic submucosal dissection (ESD). Pathological examination revealed esophageal SCC nests with intramural growth patterns.

**Outcomes:**

The first patient underwent postoperative radiotherapy, whereas the second patient did not receive any additional treatment. Both patients agreed to regular follow-up, and no tumor recurrence or metastasis was observed.

**Conclusion:**

First, not all esophageal SELs are benign, and a small number of SELs can be malignant. Second, these cases illustrate the value of newer endoscopic techniques, especially ESD. Thus, it is important to be alert when visualizing the esophagus for the possibility of a subtle SEL so that further evaluation and treatment, if necessary, can be undertaken, ideally with a less invasive approach afforded by ESD.

## 1 Introduction

Esophageal subepithelial lesion (SEL) is rare, accounting for <1% of all esophageal tumors.^[1,2]^ SELs originate from the muscularis mucosa, submucosa, or muscularis propria and are often covered with normal epithelium.^[3]^ They usually do not produce obvious clinical symptoms and are thus often incidentally found. A significant proportion of esophageal SELs are leiomyomas.^[2,4,5]^ Because only 1% of esophageal SELs are malignant, they are often overlooked or misdiagnosed.^[5]^ Therefore, the accurate and timely diagnosis of SELs is very important. Some published studies reported that endoscopic ultrasound (EUS) can be used to assess the layer of the tumor origin and to obtain tissue specimens for pathological diagnosis to determine benign and malignant tumors via EUS-guided fine-needle aspiration (FNA).^[6]^ However, EUS has some limitations in terms of lack of reliable visualization of the muscularis mucosa.^[3]^ In addition, EUS-FNA can destroy the tumor capsule, which might lead to the increase in the risk of tumor rupture and seeding.^[7]^ During recent years, with the development of endoscopic technology, endoscopic submucosal dissection (ESD) and submucosal tunnel endoscopic resection can achieve complete resection. This has led to the correct diagnosis of several malignant SELs that lacked specific endoscopic presentations.^[2,8]^

We report 2 cases of esophageal SELs removed by ESD and diagnosed as intramural squamous cell carcinoma (SCC).

## 2 Case reports

### 2.1 Case 1

A 63-year-old man was referred to our department on May 02, 2017 owing to a 2-month history of abdominal distension. He had no remarkable medical history, and physical examination indicated no positive symptoms.

**Figure 1:**
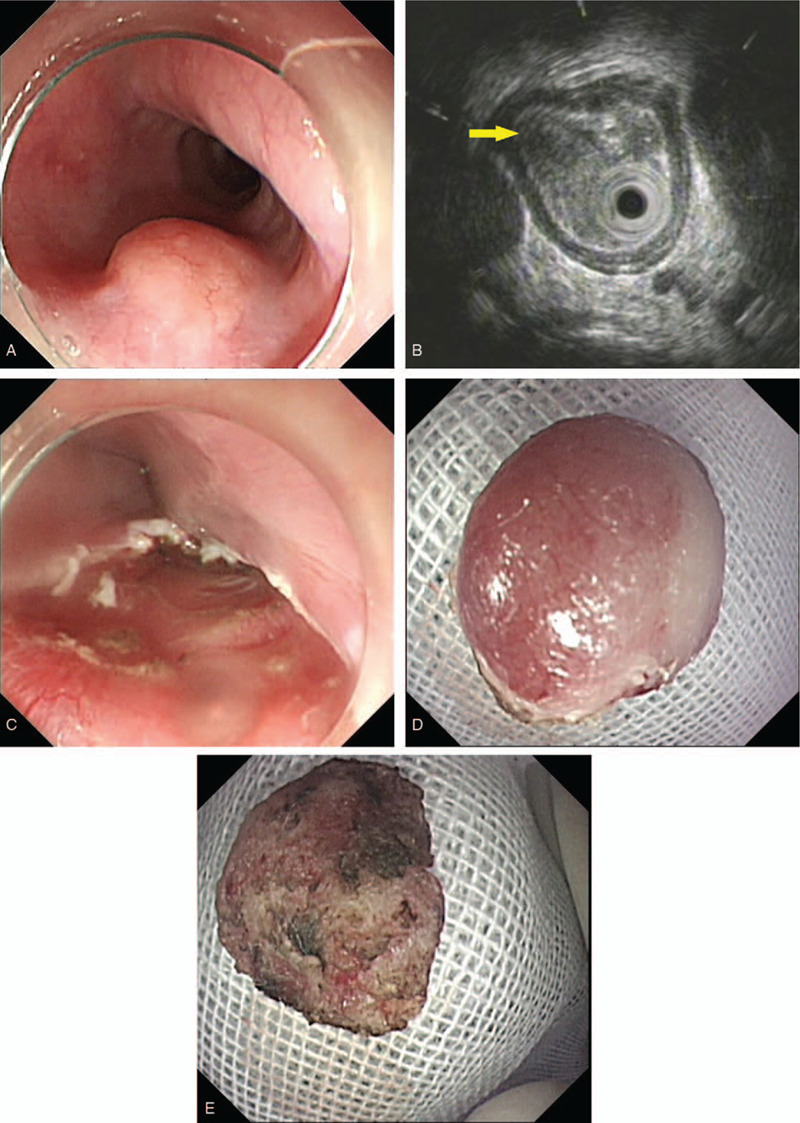
Upper gastrointestinal endoscopy in Case 1: (A) White light endoscopy shows a 1.5-cm lesion covered by smooth, normal mucosa; (B) EUS indicates well-defined, slightly heterogeneous, and hypoechoic lesions that may be derived from the muscularis mucosa or submucosa; (C) ESD, achieving en bloc removal of the lesion; (D) The size of the lesion was 1.3 × 1.0 × 0.3 cm with an intact, normal mucosa; (E) Traces of electrocoagulation can be observed on the basal surface of the lesion. ESD = endoscopic submucosal dissection, EUS = endoscopic ultrasound.

**Figure 2:**
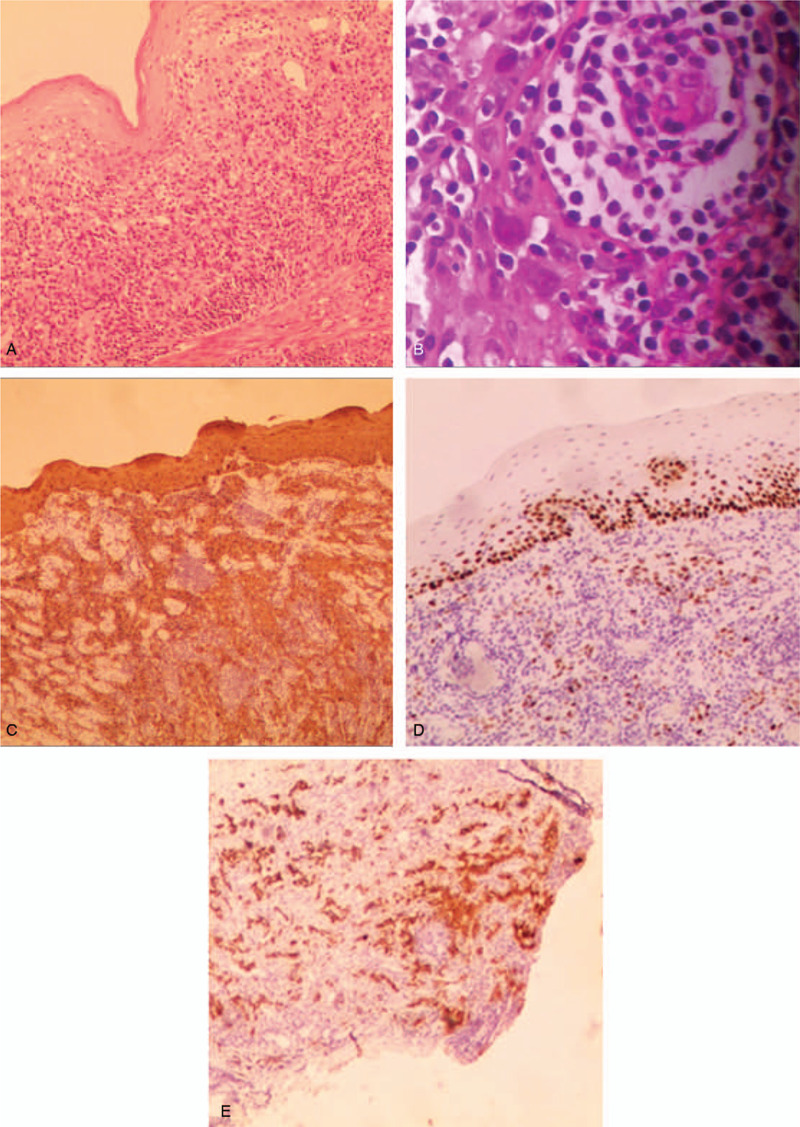
Resected specimen and histopathological examination: (A) Several scattered clusters of cells beneath a normal squamous epithelium (H&E, ×10); (B) Large cells with an increased nuclear–cytoplasmic ratio, but classification remains unclear (H&E, ×80); (C) Positive immunohistochemical staining for CK, consistent with a poorly differentiated epithelial carcinoma; (D) Positive immunohistochemical staining for P63, compatible with carcinoma cells originated from squamous cells; (E) Positive vertical margin of the tumor (CK staining, ×10). CK = cytokeratin, H&E = hematoxylin and eosin.

Upper gastrointestinal endoscopy revealed a mucosal eminence of 1.5 cm (greatest dimension) with normal overlying mucosa seen in the esophagus located at 25 cm from the central incisor (Fig. 1A). EUS revealed that it might be derived from the muscularis mucosa or submucosa. The lesion was well defined, slightly heterogeneous, and hypoechoic (Fig. 1B). ESD was performed, and the lesion was completely removed en bloc (Fig. 1C, 1D, and 1E). Histological examination using hematoxylin–eosin (H&E)-stained sections revealed some heterocysts and a normal, intact mucosal layer, although it was difficult to determine the origin and differentiation of the cells (Fig. 2A and B). Immunohistochemistry demonstrated positive staining for cytokeratin (CK), CK5/6, and P63, and approximately 20% of the cells were positive for Ki67 (Fig. 2C and D). Furthermore, stains for CD45, CD5, and CD20 were negative. Immunohistochemistry results revealed that these abnormal cells might be poorly differentiated SCC. The cancer cells were scattered in nests beneath the epithelium, and the infiltration extended from the lamina propria mucosa to the submucosa. The esophageal mucosa was intact and appeared completely normal. The basal cells were regularly shaped and normal. No obvious malignant invasion of nerves or vessels was observed. However, the vertical margin was positive (Fig. 2E).

After a multidisciplinary team discussion and based on the preference of the patient, this patient received postoperative radiotherapy (50 Gy delivered in 2 Gy per fraction) because of the vertical cutting margin was positive. Regular follow-up endoscopy, chest and abdominal CT, and serum tumor marker for 2 years did not reveal any tumor recurrence or metastasis.

### 2.2 Case 2

A 65-year-old man with a 2-month history of increasing dysphagia was admitted to our hospital on June 06, 2017. The patient's medical history was unremarkable, and physical examination revealed no characteristic features.

**Figure 3:**
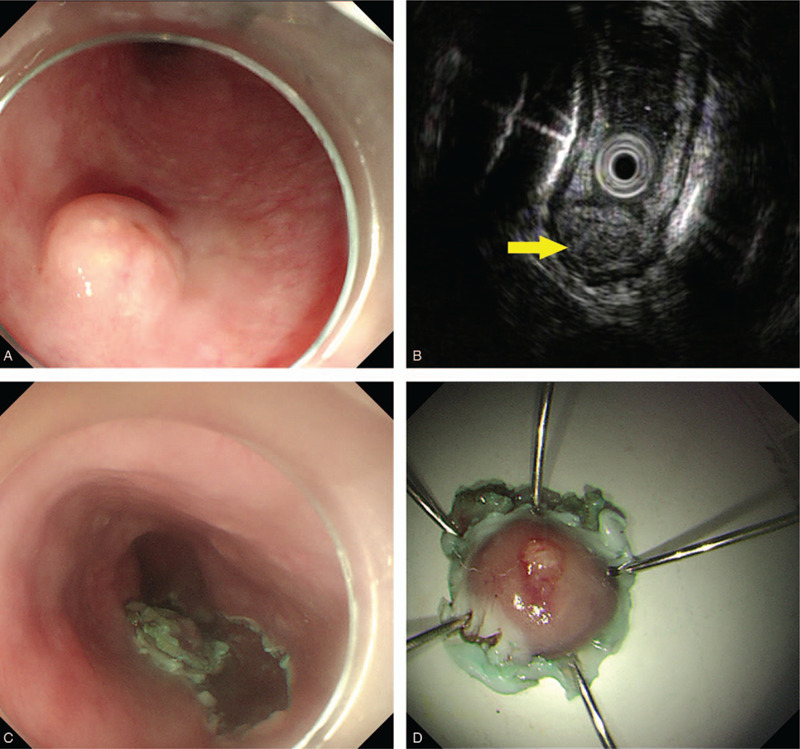
Upper gastrointestinal endoscopy in Case 2: (A) White light endoscopy shows a 1.0-cm diameter lesion covered by smooth, normal mucosa; (B) EUS shows well-defined, heterogeneous, hypoechoic lesions derived from the submucosa; (C) ESD, allowing en bloc removal of the lesion; (D) The size of the resected specimen was 1.5 × 1.5 × 1.0 cm, and the mucosa was intact. ESD = endoscopic submucosal dissection, EUS = endoscopic ultrasound.

**Figure 4:**
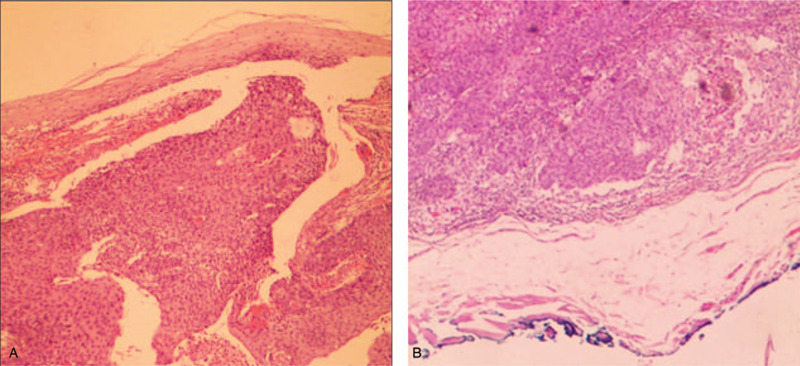
Resected specimen and histopathological examination in Case 2: (A) Cluster of atypical squamous cells observed beneath the normal epithelium (H&E, ×10); (B) Pathologically, the atypical cells indicate a moderately differentiated SCC with negative vertical margins (H&E, ×20). H&E = hematoxylin and eosin, SCC = squamous cell carcinoma.

White light endoscopy revealed a hemispherical lesion covered with smooth mucosa located at 30 cm from the central incisor (Fig. 3A). EUS demonstrated that the lesion was well defined with a hypoechoic and heterogeneous echotexture, possibly derived from the submucosa (Fig. 3B). This lesion was completely removed by ESD (Fig. 3C and D). The size of resected specimen was 1.5 × 1.5 × 1.0 cm. Histopathological examination revealed moderately differentiated intramural SCC. Esophageal mucosa was intact and completely normal. Cancer cells were present beneath the epithelium, and the infiltration extended from the lamina propria mucosa to the submucosa (Fig. 4A). No obvious infiltration was observed in nerves and vessels, and both the lateral and vertical margins were negative (Fig. 4B).

The multidisciplinary team suggested that the patient should receive regular follow-up, and postoperative treatment should be provided if any signs of recurrence or metastasis were observed. Presently, the patient's dysphagia has been considerably relieved, and no signs of recurrence and metastasis have been observed 1 year after surgery.

## 3 Discussion

Intramural growth is an extremely rare presentation of esophageal SCC, and only 5 cases have been published in the literature. McGregor et al reported the first case of esophageal intramural SCC in 1976, and the patient was treated with chemoradiotherapy instead of surgery and died 7 weeks after the final course of radiation. Autopsy revealed carcinoma cells that might have arisen from an esophageal intramural squamous epithelial cyst.^[9]^ In a 58-year-old man who underwent transhiatal esophagectomy, Von Rahden et al^[10]^ reported that postoperative pathological examination suggested SCC with an intramural growth pattern. Schmitz et al reported a case of intramural SCC located at the gastroesophageal junction with features of a benign lesion. The patient eventually underwent surgery to remove the lower third of the esophagus and the entire stomach.^[11]^ Additionally, Sonthalia et al reported a case of intramural SCC diagnosed by EUS-FNA. Subsequently, the patient was found to have a distant metastasis in the right iliac crest identified by 18-flourodeoxy glucose–positron emission tomography.^[12]^ Kishino et al reported a case of esophageal cancer with features of SET, which had already intramurally metastasized to the stomach.^[13]^

Our cases differ from these previously published reports in that the tumors were removed in both our patients by ESD. Therefore, this is the first report of esophageal intramural SCCs found and entirely resected by endoscopy. Furthermore, the 2 cases differed in the pathological findings. In case 1, the nests of SCC cells were scattered under a normal, intact mucosa and the infiltration range was from the lamina propria mucosa to the submucosa. In case 2, the nests of cancer cells were massive, and the margin of the cancer tissue was at a certain distance from the vertical margin.

According to the literature, there are 2 hypotheses about the pathogenesis of esophageal intramural SCC.^[12]^ The first hypothesis is that the tumor might have originated from the squamous epithelium in an esophageal cyst, small diverticulum, or squamous cell metaplasia in the esophageal glands. The other possibility is that cells from squamous intraepithelial neoplasia might have spread to the submucosal layers by the ducts of the submucosal glands. These cells might then have the capability to grow and infiltrate under an intact epithelium, forming a malignant carcinoma.

With the development of endoscopy and particularly of ESD, endoscopists are able to manage a disease in terms of diagnosis and resection, and this enables endoscopists to identify more lesions with caution. Although esophageal malignant SELs are very rare, some cases of malignant lesions that presented as esophageal SELs and were removed by endoscopy have been reported. Basaloid squamous cell carcinoma of the esophagus (BSCCE) is mainly visualized as an elevated lesion with a normal epithelium.^[14]^ Kim et al reported a BSCCE resected by endoscopic mucosal resection. Eventually, pathological examination revealed the involvement of vertical margin.^[15]^ Di et al^[16]^ described a case of BSCCE with special endoscopic features, including a gently rising slope on white light endoscopy, little or abnormal staining on iodine chromoendoscopy, and intrapapillary capillary loops with a small-sized avascular area on magnifying endoscopy with narrow-band imaging. This lesion was completely removed by ESD, which meant that the ability to perform en bloc resection made ESD a superior procedure for histopathological evaluation.

In addition to the abovementioned epithelial-derived esophageal malignancies, mesenchymal malignancies can occur in the esophagus. Tatsushi et al^[17]^ first reported an intraluminal polypoid form of esophageal leiomyosarcoma treated with endoscopic resection with an electric snare in 2008. Yamamoto et al^[18]^ performed en bloc resection by ESD for the therapeutic diagnosis of leiomyosarcoma in cases where several biopsy specimens could not confirm the pathology results and the tumor had rapidly grown. Additionally, esophageal GISTs can present as SELs and some of them have the potential to become malignant. Although esophageal GISTs are extremely rare, accounting for about 1% of all GISTs, the prognosis is far worse than that of gastric GISTs.^[19,20]^ Furthermore, several studies revealed that endoscopy could achieve en bloc resection in case of esophageal GISTs, and endoscopic resection is safe, effective, and minimally invasive.^[2,8,21,22]^ Moreover, several lymphomas presented as SELs and treated with endoscopic resection have been reported.^[23–27]^ In addition, according to the literature review, some other rare cases were treated with endoscopy, similar to follicular dendritic cell sarcoma and adenoid cystic carcinoma.^[28,29]^

In conclusion, esophageal intramural SCC is a rare disease, which may present with the features of SEL. It is important to be alert when visualizing the esophagus for the possibility of a subtle SEL so that further evaluation and treatment, if necessary, can be undertaken, ideally with a less invasive approach afforded by ESD.

## Acknowledgments

The authors are grateful to the 2 patients who gave their informed consent for publication.

## Author contributions

HZ and HX designed and wrote the manuscript. HZ and FL reviewed literature data. BS performed histological assessment and evaluations. HX performed the upper endoscopy and provided the endoscopic photos. HZ and FL collected the medical history of the patient. All authors critically revised the manuscript, approved the final version to be published, and agree to be accountable for all aspects of the work.

**Conceptualization:** He Zhu.

**Data curation:** He Zhu, Bing Shi, Fudong Li.

**Formal analysis:** He Zhu, Bing Shi, Fudong Li.

**Investigation:** Fudong Li.

**Methodology:** He Zhu, Bing Shi, Fudong Li.

**Resources:** He Zhu.

**Writing – original draft:** He Zhu.

**Writing – review & editing:** He Zhu, Bing Shi, Fudong Li.
